# The use of functional near infrared spectroscopy and gait analysis to characterize cognitive and motor processing in early-stage patients with multiple sclerosis

**DOI:** 10.3389/fneur.2022.937231

**Published:** 2022-08-29

**Authors:** Maria Adelia de Aratanha, Joana Bisol Balardin, Carolina Cardoso do Amaral, Shirley S. Lacerda, Tiago Abrão Setrak Sowmy, Theodore J. Huppert, Rodrigo Barbosa Thomaz, Danielli S. Speciali, Birajara Machado, Elisa Harumi Kozasa

**Affiliations:** ^1^Hospital Israelita Albert Einstein, São Paulo, Brazil; ^2^Department of Biomedical Engineering, Universidade Federal do ABC, Santo André, Brazil; ^3^Departments of Radiology and Bioengineering, Clinical Science Translational Institute, Center for the Neural Basis of Cognition, University of Pittsburgh, Pittsburgh, PA, United States

**Keywords:** multiple sclerosis, near infrared spectroscopy, functional neuroimaging, gait analysis, cognition, dual-task

## Abstract

**Background:**

Dual-task paradigms are a known tool to evaluate possible impairments in the motor and cognitive function in patients with multiple sclerosis (MS). A technique to evaluate the cortical function during movement is functional near-infrared spectroscopy (fNIRS). The evaluation of the MS course or its treatment by associating fNIRS with gait measurements may be flexible and low-cost; however, there are no feasibility studies in the literature using these combined techniques in early-stage patients with MS.

**Objective:**

To evaluate cortical hemodynamics using fNIRS and gait parameters in patients at early stages of MS and in healthy controls during a dual-task paradigm.

**Methods:**

Participants performed cognitive tasks while walking to simulate daily activities. Cortical activation maps and gait variability were used to evaluate differences between 19 healthy controls and 20 patients with MS.

**Results and conclusion:**

The results suggest an enhanced cortical activation in the motor planning areas already at the early stages of MS when compared to controls. We have also shown that a systematic analysis of the spatiotemporal gait variability parameters indicates differences in the patient population. The association of cortical and gait parameters may reveal possible compensatory mechanisms related to gait during dual tasking at the early stages of the disease.

## Introduction

Multiple sclerosis (MS) is an autoimmune inflammatory neurological disorder characterized by demyelination and axonal loss that can cause permanent damage (1, 2). It is one of the most common neurological disorders that cause disability in young adults. It is estimated that a total of 2.8 million people live with MS worldwide. The pooled incidence rate across 75 reporting countries is 2.1 per 100,000 persons/year. The mean age of diagnosis is 32 years, a highly productive stage of life when people are planning their careers and families. Women are twice as likely to live with MS than men (3).

Motor and cognitive losses are common consequences over time for people with MS (PwMS), causing deficits in attention, executive functions, information processing, and mobility (2, 4, 5); most of which impact the quality of life of PwMS, including a performance at work, social functions, and daily activities. It also comes at a high cost for society once the onset of symptoms is usually in young adulthood when people are economically active (6). The disease progression will define its severity regardless of the patient's age (2). Studies showed that gait variability could be a marker of neurological disease severity (7, 8), identify changes even at the early disease stages (9), and also help to identify gait alterations when dual tasking (10, 11). Historically, mobility is the main parameter used to evaluate the progression of MS, using the Kurtzke expanded disability status scale (EDSS). This scale ranges from 0 to 10, where 0 is normal neurological exams and 10 is death due to MS. Patients rated from 0 to 4 are considered fully ambulatory without aid (12).

Cognitive tasks were associated with increased activity mostly in the dorsolateral prefrontal cortex (DLPFC) and supplementary motor areas (SMA) (13). Functional near-infrared spectroscopy (fNIRS) is a technique that measures cortical activity as a function of oxy-hemoglobin (O_2_Hb) and deoxy-hemoglobin (HHb) (14, 15) and can be used to acquire brain data while the participant is moving (16).

The use of fNIRS in PwMS still has sparse literature; a recent review (17) showed only 11 articles and also lacks a standard protocol to evaluate dual-task (17). The first study described in the review, Hernandez et al. (18), evaluated middle-aged to elderly patients (age > 45 years, EDSS 1–6) and healthy controls (HC). Results suggest an increase in prefrontal cortex (PFC) activity for participants during dual-task trials compared to single-task ones. In another study with older adults, Chaparro et al. (19) showed that PwMS had an increased O_2_Hb concentration in the PFC when compared to HC in all tasks. In another study (20), the same group showed that PwMS had an O_2_Hb decrease in the PFC during demanding balance tasks compared to HC. This same (20) also correlates the mean of spatiotemporal gait parameters with O_2_Hb during dual tasking. In the only study to evaluate motor areas (21) during dual-task trials, Saleh et al. (21) showed that increased activation in the SMA correlated to a decrease in gait speed in PwMS compared with HC.

In this study, we created a protocol to evaluate cortical activation in both cognitive and motor areas associated with spatiotemporal gait parameters during dual tasking in early-stage PwMS and HC. Usually, at the early stages (EDSS < 4), it is difficult to capture significant changes in overall cognitive and mobility with currently available tools. However, it is also important to start early rehabilitation to improve the outcome in this population (22). Therefore, we understand that it is important to develop tools that might contribute to assessing the disease progression and intervention protocols even at early stages. The use of the dual-task paradigm in this study is an attempt to simulate how the cortical activity in PwMS would function in daily life situations when we are usually required to walk and perform attention tasks by observing our surroundings. We hypothesized that already at early stages, PwMS would show higher activation in DLPFC and SMA during dual tasking when compared to HC; however, we expected small differences in initial clinical assessments and gait variability parameters due to the low severity of the disease in our participants.

## Methods

### Participants

To participate in this study, 20 early-stage PwMS (age 35.3 ± 6.3 years, EDSS mean=1.55, range= 1–4) and 19 HC (age 35.5 ± 8.0 years) were selected. Groups were paired by age and sex, and all had completed at least bachelor's level studies. For pairing, we looked for a matched control for every PwMS enrolled in the study. PwMS were recruited from the MS center at Hospital Israelita Albert Einstein, Hospital das Clínicas de São Paulo, and Amigos Múltiplos pela Esclerose. All patients were stable for at least 6 months prior to the study and showed no clinical or radiological disease activity for the whole duration of this work.

This study was approved by the Hospital Israelita Albert Einstein ethics committee (CAAE: 7428416.2.0000.0071), and all participants signed the appropriate informed consent form according to the Helsinki declaration.

### Clinical measurements

To ensure all PwMS were at an early stage and to establish a baseline between the PwMS and HC groups in relation to their cognitive and mobility performance, all participants underwent a standard clinical evaluation for PwMS. The tests were performed by a trained neurologist and consisted of a structural MRI and the application of standardized tests such as the MS functional composite (MSFC) (23). The chosen tests evaluated cognitive and leg/ambulation function. The Paced Auditory Serial Addition Test (PASAT) is the most common test to assess cognitive performance in PwMS (23–25), where the participant listens to different numbers and must add the current one to the immediately preceding one sixty times. For ambulation assessment Timed 25 Foot Walk (T25-FW) and Timed Up and Go (TUG) were chosen, both of which are common and reliable measures to assess PwMS (23, 26–29). In the T25-FW, the participant's time was measured while walking 25 feet as fast as possible in two trials. In the TUG test, the participant's time was measured while performing the following sequence: start seated, stand up and walk 3 meters, turn around, walk back, and sit again. Both T25-FW and TUG measurements were performed two times and averaged for the final score. The EDSS of the participants was also measured as it is historically the most used neurological scale for assessing PwMS (12, 23).

During the evaluation, participants were also asked about current medication and family history of neurological diseases to take into consideration possible confounds. Results were compared with the Student's t-test test and were considered significant if *p* < 0.05.

### Experimental design

The paradigm of our study was inspired by previous work on attention-demanding locomotion in healthy older adults (30). To evaluate the cortical activity during a dual-task, the participants performed the n-back task while monitored by fNIRS. The n-back task is a well-known and reliable test to evaluate working memory. It consists of a sequence of symbols, and the participants need to indicate if the current symbol is the same as the presented n steps before, and its difficulty is increased by increasing the n steps (31, 32). It has been successfully used in PwMS as a measure of cognitive impairment (17, 33–35).

**Figure 1:**
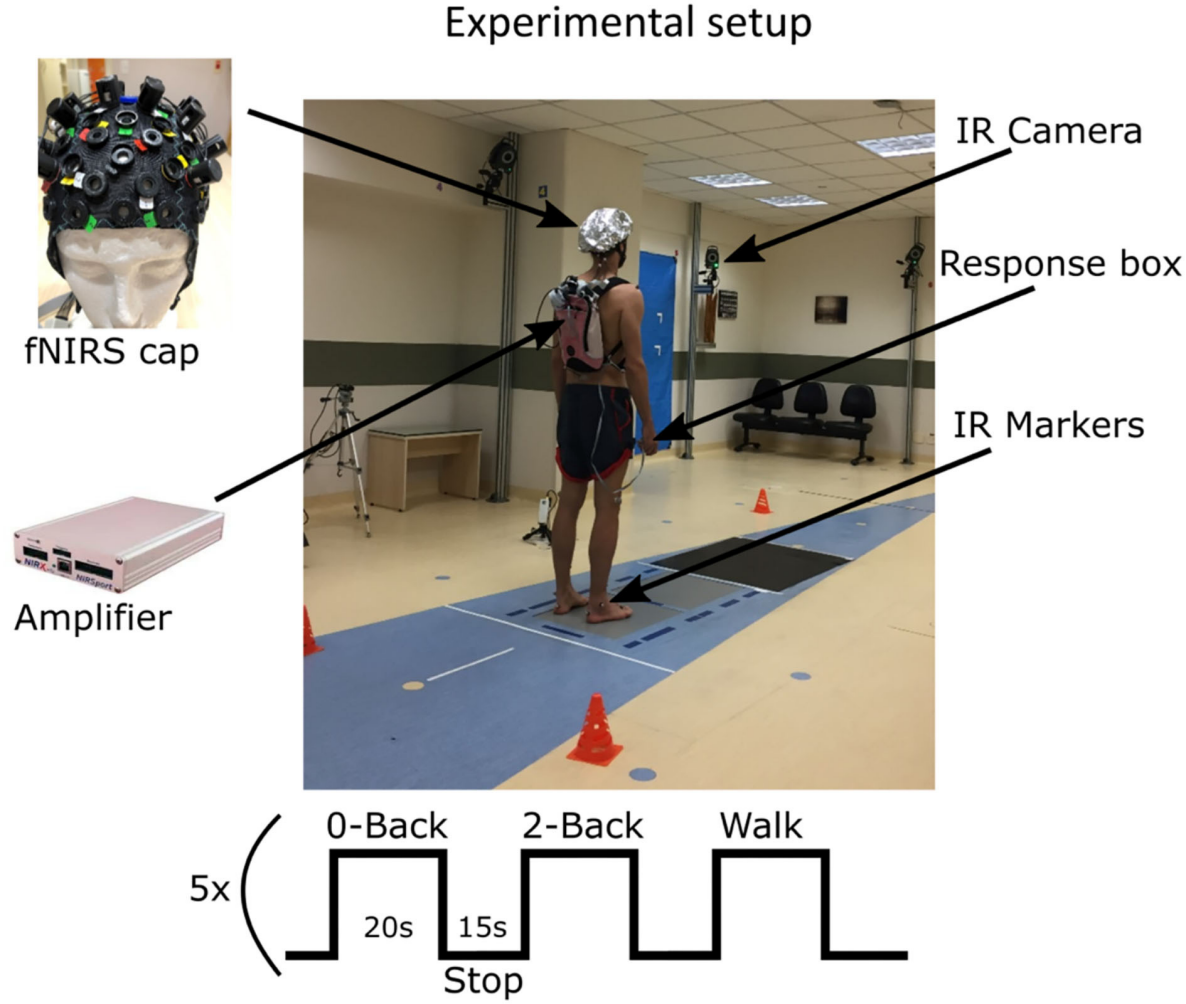
Block design and participant preparation in the gait lab. All participants walked barefoot and in gym clothes, as standard in gait analysis exams.

In this study, we used a block design with three different tasks as follows: one single task and two dual tasks, each performed five times. All participants were required to walk continuously at a comfortable pace for 20 s while performing the respective task and then stop for 15 s as shown in Figure 1. The dual tasks were auditory 0-back and 2-back tasks where the participants performed the respective cognitive test while walking. In the 0-back task, the participant heard a randomized sequence of numbers between 0 and 9 and pressed a response button as soon as the number 0 was heard. In the 2-back task, the participant heard a sequence of randomized numbers from 1 to 9 and had to press the response button if the current number was the same as the 2 positions before. For example, if they heard the sequence: 1, 5, 7, 4, 7, 8…, they had to press the button on hearing the second 7. The third and final task had no cognitive workload; the participant had only to walk and press the response box when required.

Participants' performance during 0-back and 2-back tasks was evaluated using reaction time and percentage of correct answers. Differences between groups were calculated using an ANOVA test and intra-groups using a paired t-test; both were considered significant if *p* < 0.05.

### FNIRS acquisition and analysis

**Figure 2:**
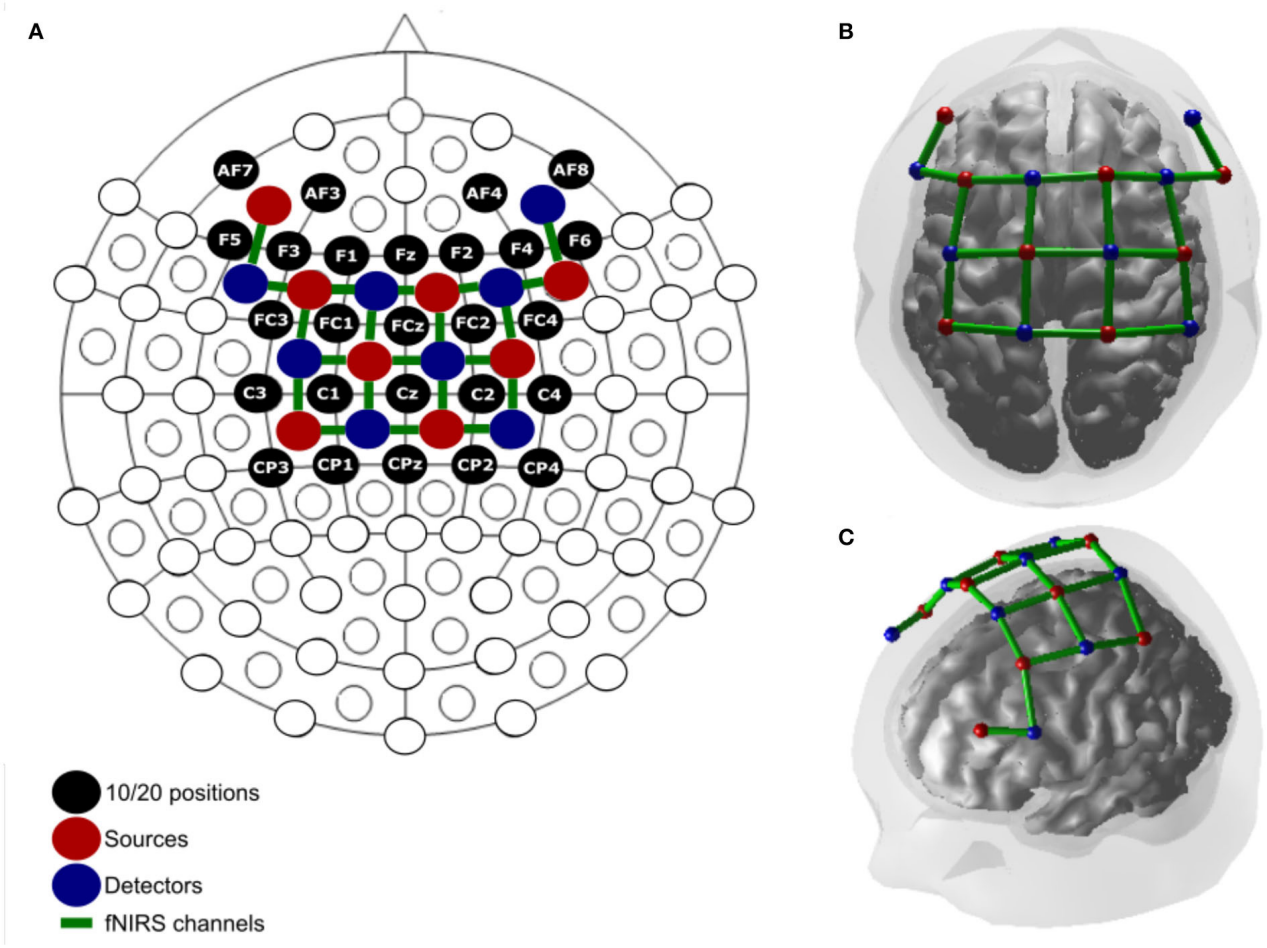
**(A)** Montage in 2D relative to the 10/20 system. **(B)** 3D superior. **(C)** Lateral view.

The cortical hemodynamic activity was acquired inside a gait lab, and the participant had the fNIRS (NIRSport, NIRx Medizintechnik GmbH, Germany) device in a backpack and could walk freely and continuously on a 5-m straight lane defined on the floor as seen in Figure 1. The fNIRS device had 8 sources and 8 detectors, forming 21 channels acquired at 7.8 Hz and wavelengths of 760and 860 nm. The 21-channel cap montage (Figure 2) used the international 10/20 system (36) and was optimized to capture activity from the primary motor cortex, SMA, premotor cortex, and DLPFC which are associated with dual tasking.

Processing and analysis of the fNIRS data were performed in the BrainAnalyzIR toolbox (37), also considering the recent consensus for gait and posture fNIRS research (16). Hemoglobin concentrations were calculated using the modified Beer-Lambert law, taking into consideration the wavelength and age to calculate the differential path length (38). The artifacts were handled with the pre-whitening algorithm of the autoregressive model using the iteratively reweighted least-squares method (AR-IRLS) described by Barker in 2013 (39). This method, available in the toolbox (37), was shown to be efficient for cleaning physiological data and motion artifacts in the fNIRS data. It consists of generalizing the statistical properties of the fNIRS signal to be more insensitive to motion and physiological artifacts, as explained in detail by Huppert in 2016 (40). The subject level response was calculated using the GLM model, applying pre-whitening and robust regression to hinder false positives (39). Group level response was calculated using the linear mixed model, using the subject as a random term (37, 41). Responses were compared using the two-tailed Student's t-test and corrected for multiple comparisons using the Benjamin-Hochberg algorithm (42); effects with *p* < 0.05 were considered significant.

The cortical activation maps were generated by mapping the significant active channels of the group level response on the cortex when testing the following three main conditions:

A. Intra-group: task (single or dual task) vs. stop (rest period) for PwMS and HC.
B. Intra-group: dual task (0-back and 2-ack) vs. single task (walk) for PwMS and HC.
C. Inter-group: PwMS vs. HC on each condition (walk, 0-back, and 2-back).

Significantly higher activity was reported in red and significantly lower activity in blue.

The walk block single task also captures noise related to the physiology of the participant's movement. When contrasting the single task vs. dual task (condition B described above), this also helps to account for the global effect caused by walking physiology, giving more precision to the cortical activity estimation due to dual tasking only.

### Gait acquisition and analysis

Gait parameters were acquired using a VICON^®^ MX 400 system (Vicon Motion Systems Ltd, UK) equipped with 10 synchronized infrared emitter and receptor cameras (Vicon MX T-Series) at a frequency of 2,000 frames per second. To hinder possible interaction between the infrared camera and the fNIRS measurement, all participants used an over cap provided by the supplier and a reflective aluminum cap. To estimate joint movement and calculate the 3D kinematics of the biomechanical model, reflective markers were glued with medical tape to the participants following the plug-in gait model protocol (43, 44). The gait tasks were performed under the supervision of a qualified physiotherapist.

All gait data were synchronized and brought to the same coordinate system using the ULTRANET^®^ device (Vicon Motion Systems Ltd, UK) followed by image reconstruction in the NEXUS^®^ software (Vicon Motion Systems Ltd, UK). From the gait cycle, 11 spatiotemporal measures were calculated relative to each task, such as stride time, cadence, step time, opposite foot off the ground, opposite foot contact to the ground, foot off the ground, single and double support, stride length, speed, and step length. These data were then imported to a customized program in MatLab (R2016a version, MathWorks, Massachusetts, USA) to calculate the coefficient of variation (CV = standard deviation/mean) for each parameter, which provides a good measure for individual gait variability once the variation is relative to the mean of that specific participant (7, 27). CV normality was verified using the Kolmogorov-Smirnov test, and group statistics were calculated using an ANOVA for each parameter for the three experimental conditions. Differences were considered significant when *p* < 0.05 after applying the Bonferroni multiple comparison correction.

## Results

### Participants

Of the 20 evaluated PwMS, 12 reported cognitive complaints (such as memory and ability to compile information). Of those, ten also reported fatigue, and two were fatigue only (without cognitive complaints). During the experiments, four of the PwMS also reported other neurological diseases such as migraine (2 participants), cephalea (1 participant), and epilepsy (1 participant), all under control. Notably, 15 PwMS reported weekly intake of vitamin D, 7 used antidepressants, 9 used immunomodulators, 11 used immunosuppressors, 16 had pulsotherapy, and 12 exercised regularly. The PwMS (EDSS mean = 1.55, range = 1–4) included in this study were diagnosed on average 5.7 ± 4.9 years ago with an average of 2.9 ± 3.11 relapses since they were diagnosed. They had a decrease in relapses in the last 2 years to an average of 0.85 ± 0.98.

In the HC group, no cognitive or fatigue complaints were reported. Moreover, three participants reported migraine. The other three used antidepressants, and eight exercised regularly.

The standard clinical tests reported no significant differences between PwMS and HC, as shown in Table 1. This suggests that even though the PwMS had a perceived loss in their cognitive function, this was not shown to be significant through the clinical tests.

**Table 1:** Comparison of the initial clinical evaluation between patients with MS and healthy controls.

**Clinical test**	**HC**	**PwMS**	***p* value**
Age	35.47 ± 8.01	35.35 ± 6.35	0.92
PASAT	45.17 ± 7.85	43.65 ± 9.14	0.59
T25FW	4.84 ± 0.49	5.14 ± 0.74	0.16
TUG	7.28 ± 1.02	7.47 ± 1.08	0.58

PASAT, paced auditory serial addition test; T25FW, timed 25-foot walk; TUG, timed up and go. The results show the mean and standard deviation for each test.

**Figure 3:**
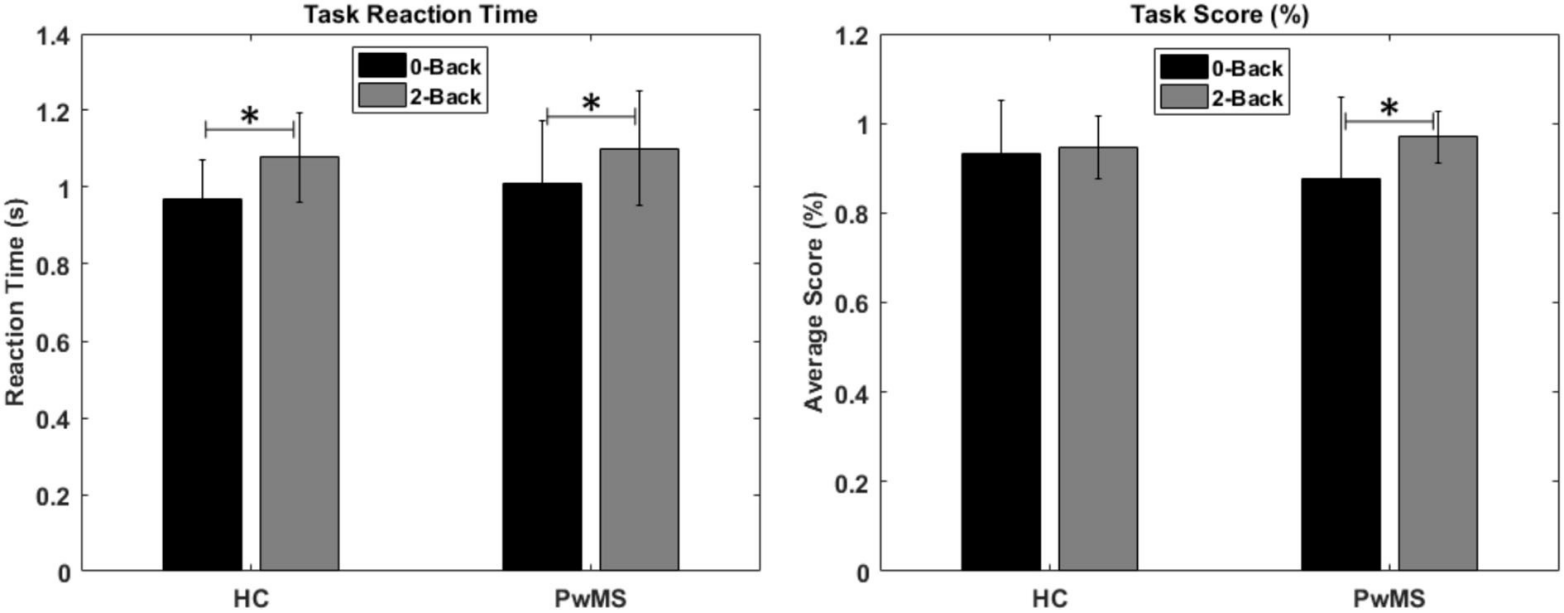
Reaction time (s) and score percentage for the 0-back and 2-back tasks in the healthy controls and people with MS. There were no significant differences between the groups. Within each group, the reaction time of the 2-back task was higher when compared to 0-back. PwMS also scored higher in the 2-back when compared to the 0-back task (*p < 0.05).

For the participant's performance, no differences were found when comparing groups. However, the reaction time was higher for both groups in the 2-back task, and PwMS scored higher in the 2-back when compared to the 0-back task (Figure 3).

### Cortical activity

**Figure 4:**
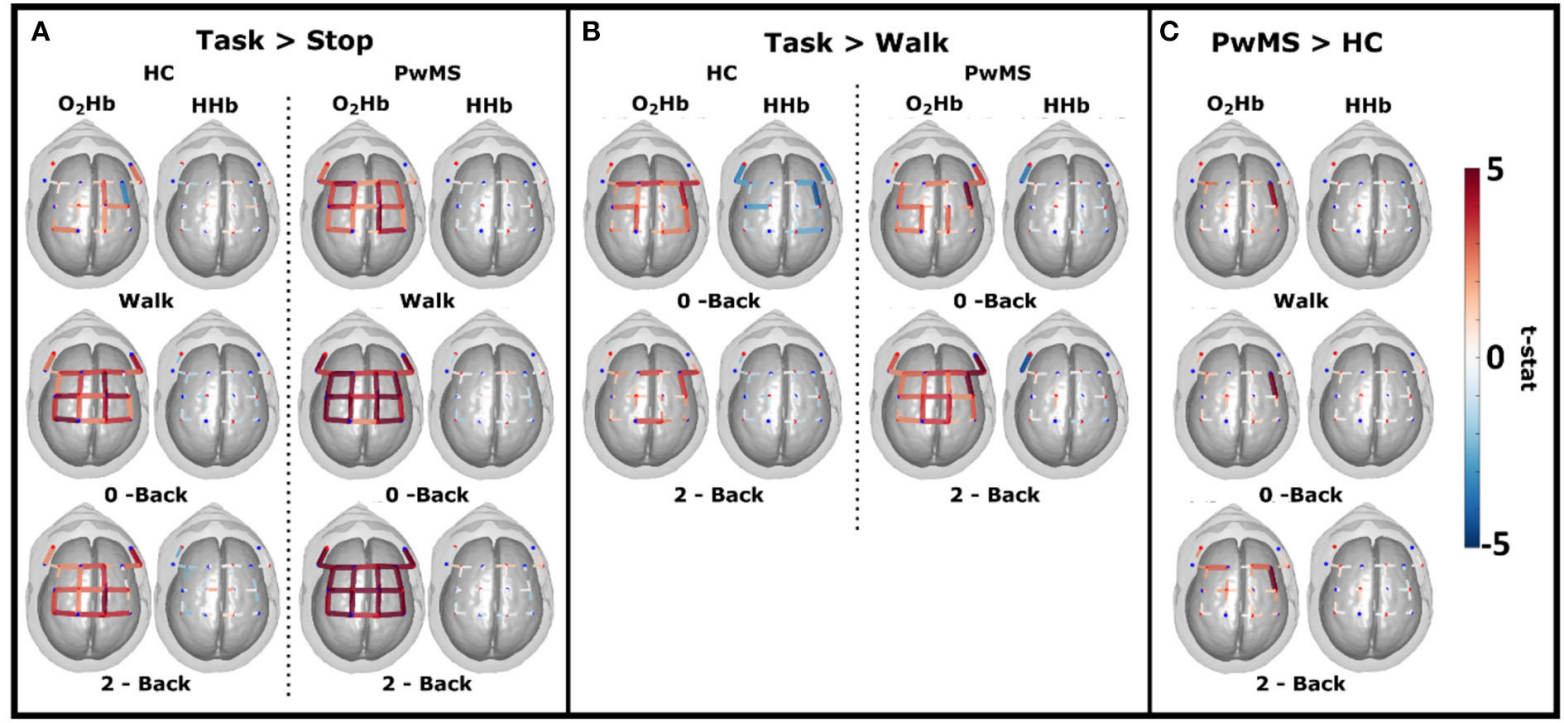
Cortical maps of all channels showing the significant t-statistic effect of oxi- and deoxyhemoglobin (O_2_Hb and HHb) from 19 healthy controls (HC) and 20 patients with MS (PwMS). **(A)** Intra-group contrast during cognitive dual tasks (0-back and 2-back) and walk relative to the rest period. **(B)** Intra-group between the cognitive dual tasks (0-back and 2-back) and walk. **(C)** Inter-group contrast during cognitive dual tasks (0-back and 2-back) and walk.

The cortical activation maps generated from group analysis in the first intra-group comparison, task vs. stop (condition A described in the “Methods” session), revealed a significant increase in the O_2_Hb in all channels for PwMS during all tasks. For HC when compared to the stop, there was a significant increase in all channels on O_2_Hb during 0-back and 2-back tasks and a more localized activity in motor areas during the walking task and a decrease in one channel from the right premotor cortex. There were no significant changes in HHb in any of the cases (Figure 4A).

In the second intra-group comparison (condition B described in the “Methods” session), dual task vs. single task, we observed a significant increase in O_2_Hb in premotor and motor cortexes in all tasks for HC and a significant decrease in HHb in dorsolateral, premotor, and motor cortexes for the 0-back. PwMS had a significant increase in O_2_Hb in premotor, motor, and right dorsolateral cortexes and a significant decrease in HHb in one channel over the left dorsolateral cortex for the 0-back task. In the 2-back task, PwMS had a significant increase in O_2_Hb in all channels and a significant decrease in HHb in the left dorsolateral area, similar to the 0-back task (Figure 4B). No significant differences were found when comparing 0-back and 2-back tasks in all groups.

In the inter-group comparison (condition C described in the “Methods” session), the cortical map revealed a significant increase in O_2_Hb in PwMS relative to HC in the right premotor cortex in the walk and 0-back tasks and bilaterally increased O_2_Hb in the premotor area for the 2-back task. No significant differences were found for HHb (Figure 4C).

### Gait analysis result

**Figure 5:**
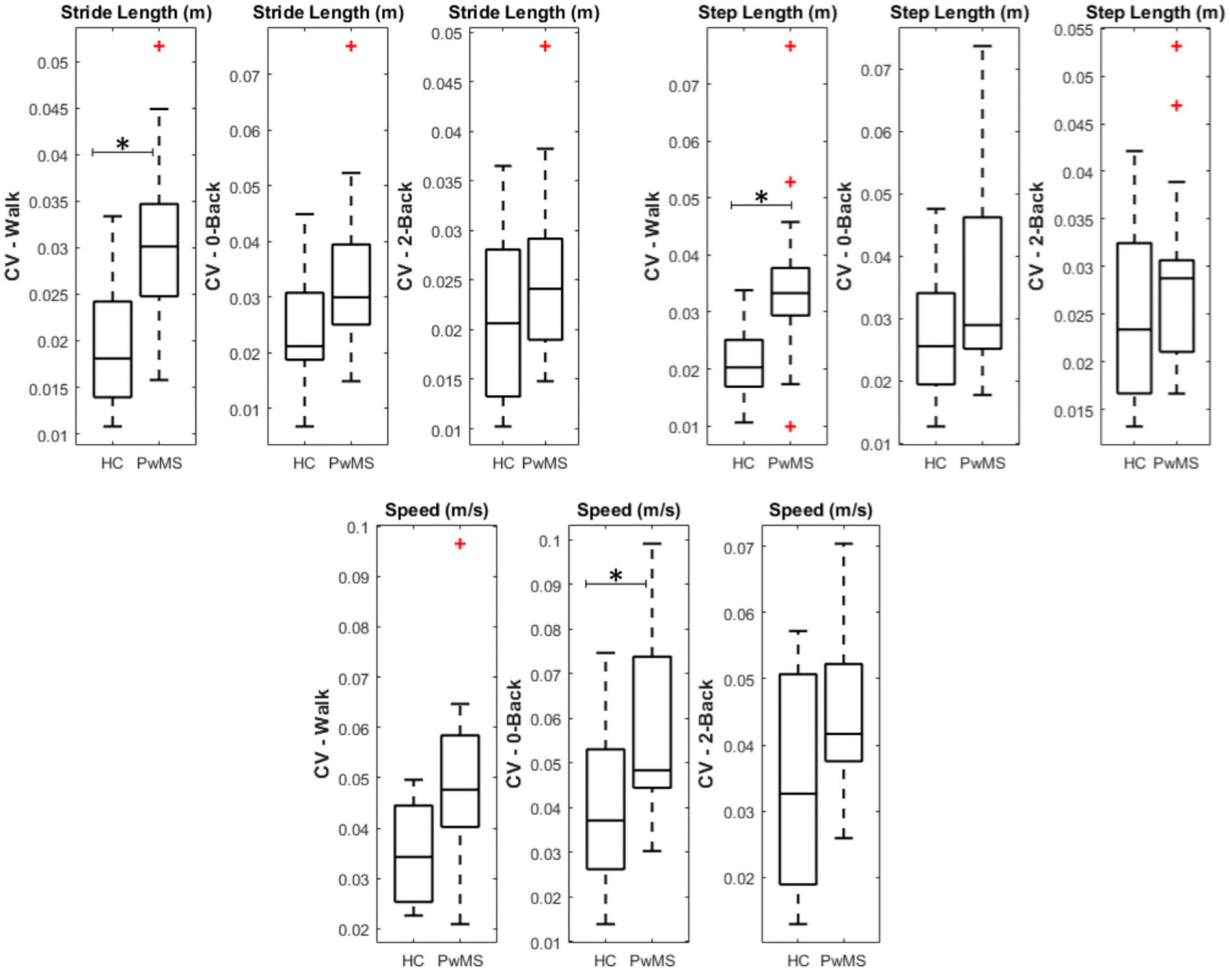
Boxplot of the significant parameters in group comparison after multi-comparison correction (*p < 0.05).

The CV analysis of the 11 gait parameters acquired during dual-tasking trials showed no significant intra-group differences. However, there were group differences between tasks in the walking task for step and stride length and speed differences in the 0-back task, as shown in Figure 5.

## Discussion

In this study, we created a protocol to evaluate the cortical activity using fNIRS and spatiotemporal gait parameters of 20 early-stage PwMS and 19 HC. We established a cognitive and mobility baseline for both groups using standard clinical tests to evaluate MS progression (PASAT, T25-FW, TUG, and EDSS). We wanted to assess whether cortical activation and gait differences could already be detected at the early stages of the disease. We used dual tasking to simulate daily activities, usually walking while performing a second task that can be more or less demanding. We used the n-back task, which is a well-known and robust way to evaluate different cognitive tasks by changing the n (31). Both HC and the PwMS group showed significantly higher reaction times in the 2-back task when compared to the 0-back, which might be interpreted as the expected higher cognitive effort (Figure 3).

Stojanovic-Radic et al. (45) also used the n-back task to evaluate cognitive activity in PwMS and HC, more specifically 0, 1, 2, and 3-back. Their results indicate that PwMS have significantly higher O_2_Hb in the PFC during easier tasks when compared to HC and a decrease in the activation in difficult conditions. They also showed a significant difference between groups in task accuracy on the easier conditions; however, the EDSS was not provided. In this study, the accuracy was higher in a more difficult condition, which might indicate an enhanced difficulty in simpler tasks. The different results could be due to the disease progression in the first study and/or that dual-tasking in early-stage PwMS might involve differences in activation for premotor but not frontal areas.

The comparison between task and stop conditions for each group (Figure 4A) shows a global effect for PwMS in all tasks. This can be interpreted as an activation of both frontal and motor areas independent of task difficulty, with the intensity being higher for more demanding tasks. The activation map is less intense for the HC group, and in the walking-only activity, there is no activation in most of the frontal channels.

The intra-group comparison (Figure 4B) indicated a significant O_2_Hb increase in the premotor and motor areas when comparing 0- and 2-back conditions to walk conditions in both groups. However, a significant increase in O_2_Hb in DLPFC was observed only for PwMS during both cognitive tasks. We also reported a significant HHb decrease in dorsolateral and premotor areas only in 0-back for HC and HHb decrease in the left dorsolateral area for PwMS in both conditions. These results are corroborated by Hernandez et al. (18), where increased O_2_Hb concentration is reported in the prefrontal areas for PwMS during dual tasking when compared to walking only.

In another study of the same group, Hernandez et al. (20) showed a decreased O_2_Hb in PwMS during dual tasking when compared to healthy older adults. The low EDSS range in this study (mean = 1.55, range = 1–4) compared to the previous studies (18, 20) (mean 3.65) can indicate alterations in the prefrontal cortex and significant differences in cognitive task performance are related to disease progression. This suggests that PwMS participating in this study preserved cognitive functions comparable to HC, which is compatible with the early stages of the disease.

Therefore, it is also interesting to evaluate cortical areas related to motor planning and motor activity during dual tasking. Saleh et al. (21) is the only study that looks only at the motor and premotor cortexes. The authors recorded fNIRS in 14 PwMS (no EDSS reported) and 14 HC during dual tasking and reported increased O_2_Hb concentration in right premotor and bilateral supplementary motor cortexes when compared to a static cognitive task for both groups. This study showed similar results, with a significant increase in O_2_Hb concentration in the premotor cortex (Figure 4C), unilateral for the easier tasks (walk and 0-back) and bilateral for the more demanding task (2-back).

In Kalron et al. (7), the authors have included more than 300 PwMS and analyzed the CV on spatiotemporal gait parameters to characterize disease progression. This study concluded that CV for step length, step time, and single support was significantly different only for moderate to severe disease stages with an EDSS above 4–5. In this investigation, we reported significant differences in the CV for three parameters (stride, step length, and speed) between groups for the 0-back condition. This suggests that systematic analysis of gait variability during dual tasking can bring additional information even for early-stage PwMS. This corroborates the findings of (9), especially when related to simple activities such as walking and low-demand dual tasks. The bilateral recruitment of premotor areas during the most demanding task (2-back) in PwMS when compared to HC could suggest a compensation mechanism in patients to maintain the same cognitive and motor performance during the task.

Monitoring the evolution of MS is mostly dependent on the use of clinical tests such as EDSS, PASAT, T25FW, and TUG, among others, and on the McDonald revised criteria (46, 47) which use structural magnetic resonance imaging that, although increasingly used around the world, still has reduced availability due to its high costs (2). fNIRS could be an interesting and low-cost way to measure brain activity for monitoring the evolution of the disease or treatment impact, especially when considering the disease in its early stages.

To the best of our knowledge, this is the first study to evaluate cortical activity in both frontal and motor areas combined with gait variability parameters in early-stage PwMS and HC. Gait variability is a known method for evaluating neurological disorders and their severity, including MS (7, 8). Combining the gait and cortical areas associated with motor control and execution can provide new information to help understand mechanisms underlying gait control in the patient population, such as compensation. This may give new opportunities to explore the disease progression or rehabilitation impact.

In this study, we used fNIRS to evaluate 19 HC and 20 PwMS. The participants performed cognitive tasks while walking to simulate daily activities. We proposed an acquisition protocol with different cognitive demand levels based on the n-back task that can be easily reproduced. We observed a higher unilateral cortical activation for easier tasks and a bilateral increase for more difficult tasks for PwMS when compared to HC in areas related to motor planning. Moreover, significant differences between groups in step and stride length, along with gait speed, were reported. The developed protocol suggests that it is possible to observe differences in early-stage PwMS. Finally, our results indicate that the association between cortical activity and gait variability could bring new light to disease progression, treatment, and rehabilitation evaluation, helping to understand the impact of daily activities on gait control in early-stage PwMS. Further studies using this protocol could also benefit from exploring the combined data as a classification tool, for example, using machine learning to classify PwMS and also the possibility to predict progression in early-stage PwMS.

## Limitations of this study

Although we designed an experiment considering the systemic and physiological changes related to movement, we are still limited in the use of new regression techniques that make use of short channels dedicated to capturing extra cortical activity and accelerometers at the probe level to quantify head motion. All data will be made available upon reasonable request.

## Data availability statement

The raw data supporting the conclusions of this article will be made available by the authors, without undue reservation.

## Ethics statement

The studies involving human participants were reviewed and approved by Comitê de Ética em Pesquisa do Hospital Israelita Albert Einstein. The patients/participants provided their written informed consent to participate in this study.

## Author contributions

MA, JB, SL, and EK contributed to the conception and design of the study. CC, TS, MA, DS, and RT did the data acquisition and database. MA, TH, and BM performed data analysis and interpretation. MA wrote the first draft of the manuscript. All authors contributed to manuscript revision, read, and approved the submitted version.

## Funding

The authors acknowledge the funding from FAPESP (2016/13104-7) and Instituto Israelita Brasileiro Albert Einstein.

## Conflict of interest

Author MA was a full-time employee of NIRx Medizintechnik GmbH during the preparation of this manuscript. The remaining authors declare that the research was conducted in the absence of any commercial or financial relationships that could be construed as a potential conflict of interest.

## Publisher's note

All claims expressed in this article are solely those of the authors and do not necessarily represent those of their affiliated organizations, or those of the publisher, the editors and the reviewers. Any product that may be evaluated in this article, or claim that may be made by its manufacturer, is not guaranteed or endorsed by the publisher.
